# New York State Dental Society

**Published:** 1872-07

**Authors:** D. Gale French


### New York State Dental Society.

FOUTH ANNUAL SESSION.

The State dental association commenced its fourth annual session in the assembly chamber, June 25th, at 10 A. M. The President, Dr. William B. Hurd, called the convention to order, and prayer was offered by Rev. Dr. Clark.

Drs. Northrop, Harvy and Merrick were appointed a Committee on Credentials. The Business Committee reported that they had, for the first time, been notified by the regular appointed essayists of the subjects of their papers, in order to report in time, viz: Subject of Dr. Leon R. Harvey's essay, "Nutrition;" Dr. W. H. Barrett, "Popular Information upon the Subject of Dentistry;" Dr. C. E. Francis, "Keeping Cavities Dry."

The morning session is to convene at 9 o'clock, and adjourn at 1½ o'clock; the afternoon session to convene at 3 o'clock and adjourn at 6½ o'clock; the evening session to convene at 8 o'clock and to adjourn upon motion. The report was accepted.

The treasurer reported the receipts for the year $670, expenditures $547,43, leaving a balance on hand on 122,57.

The Committee on Credentials reported favorably on the following members: W. H. Atkinson, W. E. Hoag, S. G. Perry (in place of Dr. Varney, deceased), W. S. Elliott, T. Le Grand Ames, Luther T. Fox, W. W. Perkins, F. D. Nellis, M. A. Newman, T. M. Snook, E. J. Sine, B. F. Schuyler, C. W. Stanton, W. C. Barrett.

Dr. M. H. Webb, of Lancaster, Pa., delegate and member of the Pennsylvania State Dental Society, was duly elected an honorary member.

The President then read his annual address, and in it alluded to the death of Dr. Whitney. Dr. W. A. Barrett referred in glowing terms to the character of the deceased.

A committee of three was appointed to draft suitable resolutions on the death of Dr. Whitney; also a similar committee on the death of Dr. Varney. The reports of the several district societies were read and ordered filed. The Board of Censors recommended the following doctors for deplomas: Edward J. Sine, Rochester; Fred. E. Perry, Norwich; C. W. Stanton, Lancaster. [Laid on table.]

The Publication Committee reported that they had not deemed it advisable to publish the proceedings of last year, as they were not large enough, and that they had concluded to publish the transactions of two years (this and last) in one volume. Accepted.

The committee to procure a form of certificate for the several district societies submitted a report, which, afier discussion, was adopted.

The committee appointed last February to oppose the extension of the Goodyear rubber patent, presented a report which was accepted, and the committee discharged.

Dr. Jarvis offered the following which was adopted:

Resolved, That the attention of the district dental societies be called to the fact that this soeiety has interpreted the law to designate the name of the various district societies as "the district dental society of the —— judical district."

Drs. Kinsley and Perkins were appointed to fill vacancies on the Committee on Ethics.

Recess.

The society reconvened at 3 o'clock.

The afternoon session was devoted to the reading of the essays of Drs. Harvey and Francis, and a discussion of the same.

EVENING SESSION.

The society met again at 8 o'clock, Dr. Hurd in the chair. Drs. Brownson, Miller and Atkins were appointed a Committee on Prize Essays.

The committee appointed at the morning session to prepare suitable resolutions to the death of Drs. Whitney and Varney, submitted reports which were adopted.

An obituary sketch of Dr. Whitney's life was read by Dr. Freeman.

Dr. Ambler read a brief address on the subject of perpetuating the memory of deceased members of the society, and offered a resolution directing the Publication Committee to procure suitable likenesses of Dr. Stevens, Snow, Whitney and Varney, to be bound with the volume of transactions; also, that a memorial page, in the records of this society be properly engrossed with a likeness of each in the center. Adopted.

A motion was adopted that the annual meeting end at the close of the afternoon session to day.

Adjourn until 9 o'clock this morning.

SECOND DAY'S SESSION.

The society met at 9 o'clock, President Hurd in the chair. Prayer was offered by the Rev. Mr. James.

The Committee on By-Laws submitted a report which was adopted.

The report of the Censors was also received and adopted.

Dr. Barrett read an essay on "Popular Information on the subject of Dentistry."

The Secretary reported accounts amounting to $131,50, which were ordered paid.

A motion was adopted to pay the Secretary $100 as a remuneration for past services.

The society proceeded to the election of officers for the ensuing year with the following result.

President—C. A. Marvin, of Brooklyn.

Vice President—W. C. Barrett, of Warsaw.

Recording Secretary—Charles Barnes, of Syracuse.

Corresponding Secretary—S. A. Freeman, of Buffalo.

Treasurer—A. C. Hawes, of New York City.

Drs. C. D. Cook, of the second district, and S. B. Palmer, of the fifth district, were elected censors in the place of Drs. Hurd and Wascott, whose terms of office have expired.

The following were elected permanent members of the society: S. G. Perry, D. F. Harvey, W. H. Atkinson, D. Monroe, J. C. Austin, S. M. Snooks.

The following were chosen delegates to the American National Association at Niagara Falls: W. C. Barrett, L. F. Harvey, S. A. Freeman, W. H. Young, W. Jarvie, Jr.

The following were elected delegates to the Canada dental association: W. C. Barrett, G. C. Daboll, W. H. Atkinson.

J. G. Ambler was elected delegate to the Pennsylvania Dental Association.

Drs. Hill and McCall were appointed a committee to conduct the president elect to the chair, and introduce him to the society. The President returned his thanks for the compliment conferred upon him, and pledged himself to act honorably towards all, and be governed by the rules and by-laws.

The President then announced the following standing committees.

Arrangements—J. C. Austin, of Albany; J. A. Perkins, of Albany; S. D. French, of Troy.

On Business—C. E. Francis, H. G. Merick, W. W. Perkins.

Publication—L. W. Rogers, S. A. Freeman, S. H. McCall.

By-Laws—William Carr, A. L. Northrop, A. M. Holmes.

Ethics—E. A. Bogue, W. A. Brownson, G. C. Deboll.

The Committee of Arrangements were authorized to employ a stenographer for the next session.

The society then adjourned *sine die*.

The Mad River Valley Dental Association held its regular semi-annual meeting at the office of Dr. Geo. L. Paine, Xenia, Ohio, on the 14th of May last. In the absence of the President, Vice President and Secretary, Dr. Watt was chosen President *pro tem.*, and Dr. Paine Secretary *pro tem.* The retiring President having ommitted the appointment of an Executive Committee at the last regular meeting, there was no programme prepared; and in the absence of which no discussions were participated in. Committee on Membership was then appointed by the Chair; Dr. Bradley and Harlan constituting said Committee. Dr. Geo. Paine moved Dr. D. Gale French be admitted as a member of this association; carried. The association then proceeded with the election of officers for the ensuing year, with the following result: President, Dr. Geo. Watt, Xenia, Ohio; Vice President, Dr. C. Bradley, Dayton, Ohio; Recording Secretary, Dr. D. Gale French, Xenia, Ohio; Corresponding Secretary, Dr. J. S. Stipp, Sidney, Ohio. Drs. Geo. J. L. Paine, C. Bradley, and S. H. Harlan, were appointed an Executive Committee by the Chair; who presented the followiug programme for general discussion for the next regular meeting: 1st. Treatment of Aching Teeth and Exposed Nerves Preparatory to Filling, to be presented by Dr. Bradley. 2d. On the Best Mode of Separating Teeth Preparatory to Filling Proximal Cavities. 3d. Treatment of Periostitis. 4th. What Form of Plates Will Best Insure a Natural Expression of the Features. 5th. Miscellaneous Business. The President then appointed the following to prepare essays to be read at the next regular meeting: Drs. Geo. L. Paine, D. Gale French, J. A. Stipp, and Geo. W. Keeley. Springfield on motion of Dr. Harlan, was selected for the next meeting, to convene on Teusday, the 24th day of September next. The following were then elected delegates to the American Dental Association: Drs. C. Bradley, Dayton, Ohio; D. Gale French, Xenia, Ohio; S. H. Harlan, Mechanicsburgh, Ohio; Geo. L. Paine, Xenia. Ohio; A. O. Rawls, Connersville, Indiana; J. N. Welch, Eaton, Ohio; C. R. Sabin, Lima, Ohio, and J. Frank McGinnis, Bellefountain, Ohio. And the association, in absence of further business, adjourned to meet as above.

D. Gale French.
Recording Secretary.

